# Running exercise protects oligodendrocytes in the medial prefrontal cortex in chronic unpredictable stress rat model

**DOI:** 10.1038/s41398-019-0662-8

**Published:** 2019-11-28

**Authors:** Yanmin Luo, Qian Xiao, Jin Wang, Lin Jiang, Menglan Hu, Yanhong Jiang, Jing Tang, Xin Liang, Yingqiang Qi, Xiaoyun Dou, Yi Zhang, Chunxia Huang, Linmu Chen, Yong Tang

**Affiliations:** 1 Laboratory of Stem Cells and Tissue Engineering, Chongqing Medical University, Chongqing, 400016 People’s Republic of China; 2 Department of Physiology, Chongqing Medical University, Chongqing, 400016 People’s Republic of China; 3 Department of Radioactive Medicine, Chongqing Medical University, Chongqing, 400016 People’s Republic of China; 4 Department of Histology and Embryology, Chongqing Medical University, Chongqing, 400016 People’s Republic of China; 5 Institute of Life Science, Chongqing Medical University, Chongqing, 400016 People’s Republic of China

**Keywords:** Depression, Neuroscience

## Abstract

Previous postmortem and animal studies have shown decreases in the prefrontal cortex (PFC) volume and the number of glial cells in the PFC of depression. Running exercise has been shown to alleviate depressive symptoms. However, the effects of running exercise on the medial prefrontal cortex (mPFC) volume and oligodendrocytes in the mPFC of depressed patients and animals have not been investigated. To address these issues, adult male rats were subjected to chronic unpredictable stress (CUS) for 5 weeks, followed by treadmill running for 6 weeks. Then, the mPFC volume and the mPFC oligodendrocytes were investigated using stereology, immunohistochemistry, immunofluorescence and western blotting. Using a CUS paradigm that allowed for the analysis of anhedonia, we found that running exercise alleviated the deficits in sucrose preference, as well as the decrease in the mPFC volume. Meanwhile, we found that running exercise significantly increased the number of CNPase^+^ oligodendrocytes and Olig2^+^ oligodendrocytes, reduced the ratio between Olig2^+^/NG2^+^ oligodendrocytes and Olig2^+^ oligodendrocytes and increased myelin basic protein (MBP), CNPase and Olig2 protein expression in the mPFC of the CUS rat model. However, running exercise did not change NG2^+^ oligodendrocyte number in the mPFC in these rats. These results indicated that running exercise promoted the differentiation of oligodendrocytes and myelin-forming ability in the mPFC in the context of depression. These findings suggest that the beneficial effects of running exercise on mPFC volume and oligodendrocytes in mPFC might be an important structural basis for the antidepressant effects of running exercise.

## Introduction

Major depression disorder (MDD) is a very common mental and emotional disorder, and its high recurrence rate leads to a decline in quality of life and high mortality^1^. The mPFC has been deemed to be one of important brain areas vulnerable to stress exposure and associated with MDD^2,3^. Findings from Ansell et al. indicated that cumulative exposure to adverse life events was associated with a smaller mPFC volume^4^. Neuroimaging studies have demonstrated that PFC volume reductions were associated with MDD, especially reductions in the mPFC or its subregion^3,5–7^. Many other clinical studies also indicated that the mPFC volume was closely related to depression and might be an indicator of the illness burden, medication status and treatment response^2,6,8,9^. However, in all previous human studies, the researchers had obtained indirect information from neuroimaging, and they could not accurately measure the mPFC. Therefore, it remains necessary to accurately investigate the mPFC volume change in depression.

Postmortem studies have provided morphometric evidence that reductions in glial cell density may be one of the important factors contributing to the decrease in frontal volume in MDD patients^10,11^. Hamidi et al. found that glial cell reduction in the amygdala in MDD patients was primarily due to oligodendrocytes^12^. Oligodendrocytes are one type of central nervous system glial cells present in vertebrates. Mature oligodendrocytes can produce a laminated, lipid-rich myelin sheath that wraps neuronal axons to maximize the speed of action potential conduction^13^. Postmortem studies have provided evidence that the oligodendrocyte density and the expression of oligodendrocyte-related genes were significantly reduced in the PFC of MDD patients^14–16^. Meanwhile, evidence implicating decreased oligodendrocyte components in MDD was paralleled by several studies in rodent models^17–19^. Rodents exposed to chronic stress showed a decrease in the proliferation of oligodendrocytes in the PFC; however, this pattern could be reversed with antidepressant treatment^20,21^. Moreover, clemastine, which has been shown to enhance myelination under demyelinating conditions, successfully reversed social avoidance behavior and impaired myelination in the mPFC of adult socially isolated mice^22^. It can be seen that oligodendrocyte reduction in the mPFC is an important pathological change in depression, and further research will be helpful for exploring new antidepressant strategies. However, no study has immunolabeled oligodendrocytes with specific stage markers, especially the critical oligodendrocyte stages of oligodendrocyte progenitor cells (OPCs) and mature oligodendrocytes and then accurately quantitated the oligodendrocytes in the mPFC in MDD subjects or animal models.

Running exercise has been proven to be an effective behavioral antidepressant therapy. A large number of clinical studies have demonstrated that physical exercise could increase resilience to stress in individuals, effectively treat MDD patients of different ages and significantly decrease the relapse rate^23–25^. In rodent studies, both voluntary and forced running exercise could improve depression-like symptoms^26,27^. As mentioned above, the reduced volume of the mPFC and the oligodendrocyte lesions in the mPFC are important pathological changes in MDD. Coincidentally, some clinical studies have indicated that physical exercise could increase the PFC volume in normal individuals or schizophrenia patients^28–30^ and preserve the PFC volume against aging^31^. In addition, animal studies have indicated that physical exercise could increase the oligodendrocyte proliferation in the PFC of animal models^32^, while other studies have demonstrated that physical exercise showed the opposite effect on oligodendrocyte proliferation but enhanced oligodendrocyte differentiation^33^. Treadmill running has been reported to alleviate the depressive-like behavior in several animal models useful for the study of depression^27,34–37^. Our previous studies found that running exercise had positive effects on the myelinated fibers of white matter and the oligodendrocytes of the hippocampus in rats exposed to CUS^36,37^. However, the effects of running exercise on the mPFC volume and the oligodendrocytes in the mPFC in the context of MDD are unclear. Therefore, in the present study, a CUS-induced rat model, an animal model useful for the study of depression, was used and subsequently administered 6 weeks of treadmill running. The effects of treadmill running on the mPFC volume and the oligodendrocytes of the CUS rats were investigated with stereological methods, immunohistochemistry and western blotting. We found that running exercise could reverse depressive-like behaviors and volume loss in the mPFC and promote oligodendrocyte differentiation and myelination of the mPFC in the CUS-induced rat model of depression. Our findings might provide structural bases for the exercise-induced treatment of depression.

## Materials and methods

See the Supplementary Materials and Methods for more details.

### Animals

Sixty male Sprague–Dawley rats (Chongqing Medical University, Chongqing, China) were housed under a 12-h light/12-h dark cycle at a constant temperature (22 °C) with free access to food and water. After 2 weeks of acclimation, all rats were randomly divided into a nonstressed group (*n* = 23), stressed group (*n* = 17) and stressed + running group (*n* = 20). The behavioral experiments and tissue processing were performed with the rats following 5 weeks of CUS and 6 weeks of running exercise. During the animal experiment, the animals in each group were treated by the investigators without blinding. All experiments were approved by the Animal Care Committee of the Chongqing Medical University.

### CUS paradigm

For CUS, the rats in the stressed group and stressed + running group were housed with one rat per cage and exposed to two to three stressors per day for 5 weeks. The protocol was adapted from our previous reports^36,37^.

### Treadmill running

After the CUS intervention, rats in the stressed + running group were scheduled for treadmill running using a horizontal motorized treadmill for 6 weeks, 5 days per week, 20 min per day. During the first week, the rats ran at a speed of 10 m/min on the first day, followed by an increase of 2 m/min per day. For the remaining 5 weeks, the speed was maintained at 20 m/min. This treadmill running pattern has been used successfully in our previous studies^36,37^.

### Behavioral tests

The body mass of each rat was recorded and the sucrose preference test (SPT) was assessed during the same time frame each week. The elevated plus maze test was performed in the last week of running exercise.

### Perfusion and tissue processing

During the following processes, all the experiments and data analyses were performed blind to treatment conditions. After the behavioral testing, under deep anesthesia with an i.p. injection of 1% sodium pentobarbital (4 mL/kg body weight), five rats from each experimental group were randomly selected and perfused transcardially with 4% paraformaldehyde. Brains were removed and split into two hemispheres by a midsagittal section. The right or left hemisphere from each rat was sampled at random and coronally sectioned into 60 μm sections on a cryostat microtome (CM1860, Leica). The sections were kept in anatomical series. From the sections containing the mPFC, every 6th section was sampled in a systematic-random manner with 15 sections per series on average. In the end, six sets of sampled sections were acquired.

### Cresyl violet staining and volume estimation

**Fig. 1:**
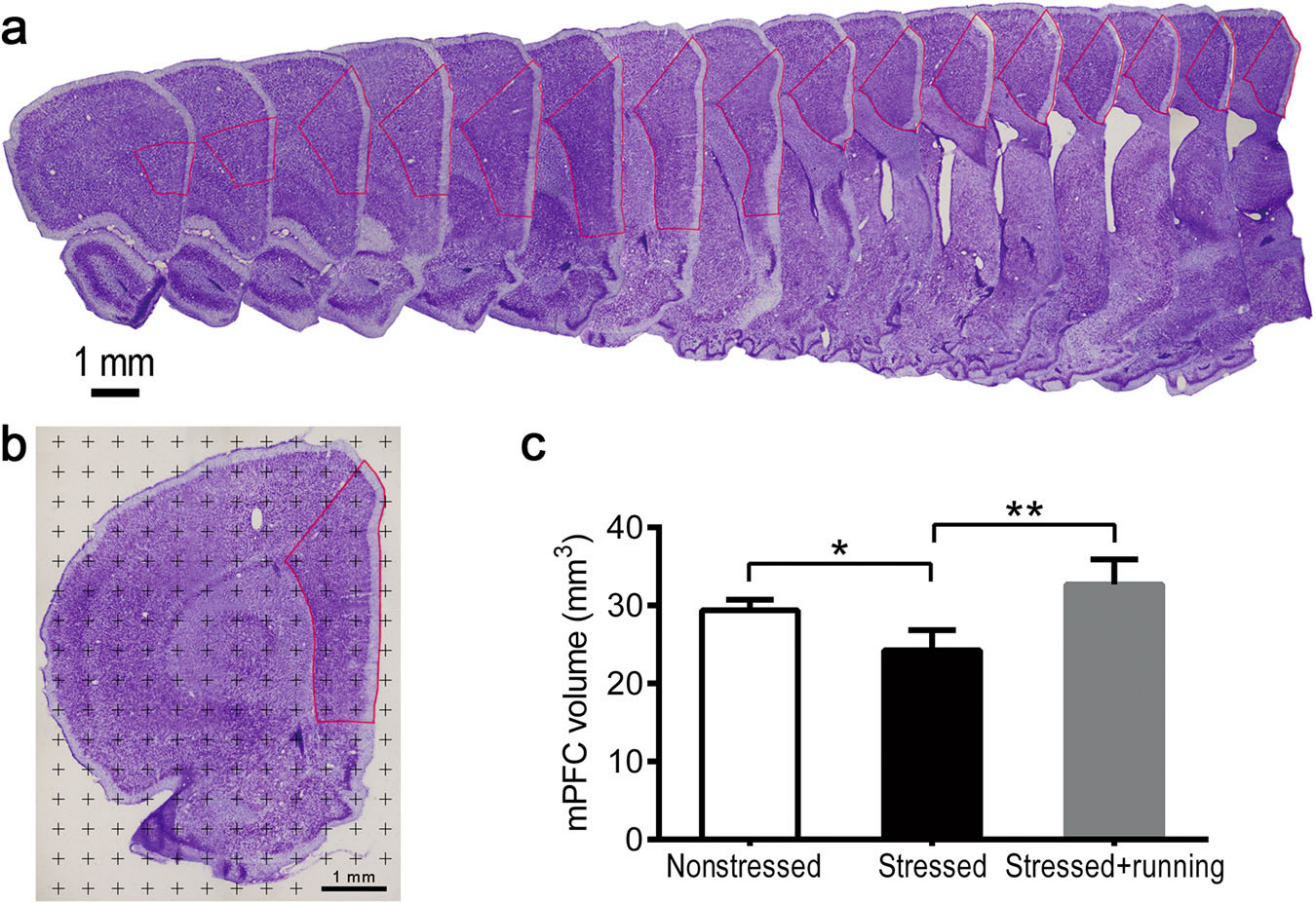
Illustrations of the methods to quantify the mPFC volume and the effects of running exercise on the total volume of the mPFC of CUS rats. **a** An illustration of the mPFC boundary after cresyl violet staining. The area within the red lines was the mPFC. Scale bar = 1 mm. **b** An illustration of the method used to estimate the total volume of the mPFC. The points located inside the red line were counted. Scale bar = 1 mm. **c** Total volume of the mPFC in the nonstressed group, stressed group and stressed + running group (mean ± SD, *n* = 5 per group). *p < 0.05, **p < 0.01. mPFC medial prefrontal cortex.

One set of sampled sections was randomly chosen. The sampled sections were stained with cresyl violet to help delineate the boundary of the mPFC. The mPFC of rats is composed of prelimbic (PL), infralimbic (IL) and anterior cingulate (ACC) cortices^38^. Under a low-magnification objective lens (4×), the boundary of the mPFC was delineated according to the description in Cerqueira *et al*.^38^ and the atlas of Paxinos and Watson^39^ (Fig. 1a). The total volume of the mPFC was measured using Cavalieri’s principle^36^ (Fig. 1b).

### Immunohistochemistry and stereological cell counting

Two separate sets of serial sections containing the mPFC from each group of rats were chosen and immunoreacted with mouse anti-NG2 antibody (ab50009, Abcam, USA) and mouse anti-CNPase antibody (ab6319, Abcam, USA), respectively, for the stereologic analyses of the total numbers of OPCs and mature oligodendrocytes in the mPFC. The optical fractionator^40^ was used to estimate the total numbers of NG2^+^ and CNPase^+^ cells in the mPFC (Supplementary Fig. 1a, b).

### Immunofluorescence and analysis

From one set of sampled sections, every second section was sampled again in a systematic-random fashion resulting in two series of sections with eight sections per series on average. The two series of sections were labeled with Olig2/NG2 antibodies against the OPCs and MBP antibodies against the myelin sheaths. Sixteen randomly chosen fields were used for cell counting in each rat. Quantification of MBP immunostaining was performed using NIS-Elements 4.3.

### Western blotting

Rats (eight per group) were randomly chosen and anesthetized with sodium pentobarbital, and mPFC tissue was rapidly removed and homogenized, and proteins were extracted using a RIPA lysis buffer with 1% PMSF solution (Beyotime Biotechnology, China). After protein determination using a BCA kit (Beyotime Biotechnology, China), sodium dodecyl sulfate–polyacrylamide gel electrophoresis and western blotting were carried out for detecting the protein levels of MBP, NG2, CNPase and Olig2. Quantification of band intensity was analyzed using Image Lab software (version 5.2.1).

### Statistics

All statistical analyses were performed using SPSS 19.0. The Shapiro-Wilk test was used to evaluate whether the data were normally distributed. The Levene’s test was used to evaluate whether the variances were similar among the groups. Then the data from the body weight were analyzed using repeated measures analysis of variance (ANOVA). For the remaining data, if they were normally distributed and had similar variance among groups, one-way ANOVA followed by LSD post-hoc test were used for analysis; otherwise, Kruskal-Wallis test was adopted for analysis. A *p* value <0.05 was adopted as the level of significance. The coefficient of error (CE) of the mPFC volume was calculated according to Gundersen *et al*.^41^. The CE of the NG2^+^ cells and CNPase^+^ cells in the mPFC were calculated according to the method described by Schmitz and Hof^42^. Sample size for each experiment was chosen based on previous experience and aimed to detect at least a *p* < 0.05 in the different tests applied. No animals were excluded from the current study.

## Results

### Running exercise alleviated anhedonia induced by CUS

**Fig. 2:**
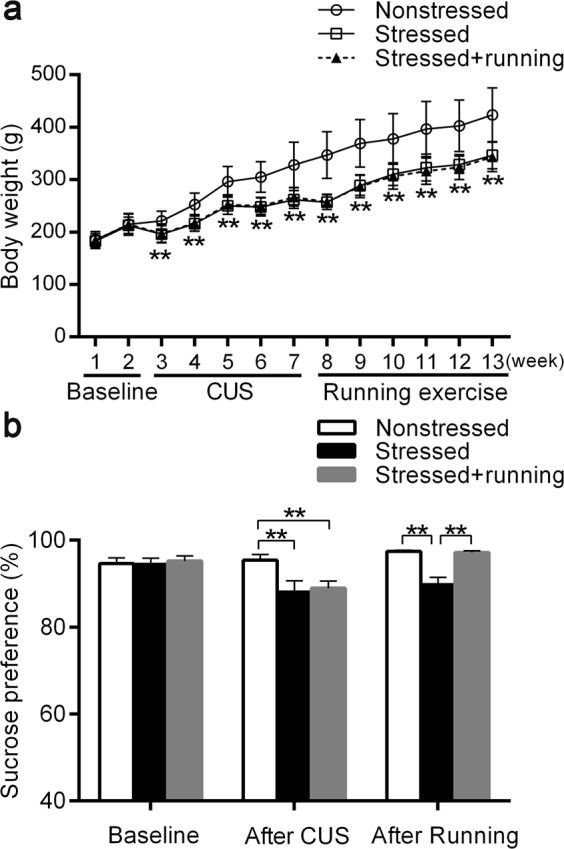
The effects of running exercise on the depression-like behaviors of CUS rats. **a** The body mass changes in nonstressed group (*n* = 23), stressed group (*n* = 17) and stressed + running group (*n* = 20) at different stages (mean ± SD). **p < 0.01, when the body mass of the stressed group and stressed + running group are compared with that of the nonstressed group. **b** The sucrose preferences of nonstressed group (*n* = 23), stressed group (*n* = 17) and stressed + running group (*n* = 20) at different stages (mean ± SD). **p < 0.01. CUS chronic unpredictable stress.

The body weights for each group of rats is shown in Fig. 2a. Rats in the stressed group and stressed + running group showed persistently significantly lower body weight from the second week of CUS intervention than the rats in the nonstressed group (repeated measures ANOVA *F*_(2,57)_ = 38.63, *p* *<* 0.001; post-hoc, *p* < 0.001 for stressed vs. nonstressed, *p* < 0.001 for stressed + running vs. nonstressed, Fig. 2a). After the CUS intervention, rats in the stressed group and stressed + running group showed a significantly lower sucrose preference than the rats in the nonstressed group rats (one-way ANOVA *F*_(2,57)_ = 12.537, *p* *<* 0.001; post-hoc, *p* < 0.001 for stressed vs. nonstressed, *p* < 0.001 for stressed + running vs. nonstressed, Fig. 2b). As expected, 6 weeks of running exercise significantly increased the sucrose preference of the rats that had been stimulated by the CUS (one-way ANOVA *F*_(2,57)_ = 12.537, *p* *<* 0.001; post-hoc, *p* < 0.001 for stressed vs. nonstressed, *p* < 0.001 for stressed + running vs. stressed, Fig. 2b). In the elevated plus maze test, the time in close arms (s) of stressed + running group was significantly shorter than that of the stressed group (one-way ANOVA *F*_(2,57)_ = 6.079, *p* = 0.004; post-hoc, *p* *=* 0.002 for stressed + running vs. stressed). There were no significant differences among the three groups of rats for other parameters assessed in the elevated plus maze test (Supplementary Table 1).

### Running exercise protected against mPFC volume loss induced by CUS

In the present study, the stereological method was used to accurately estimate the mPFC volume in the three groups of rats. The mean points counted in the mPFC in the nonstressed group, stressed group and stressed + running group were 199, 164 and 221 with CEs of 7.1%, 7.8% and 6.7%, respectively. The mean mPFC volumes were 29.37 ± 1.39 mm^3^ in the nonstressed rats and 24.25 ± 2.60 mm^3^ in stressed rats, representing a significant decrease of 17.43% induced by CUS (one-way ANOVA *F*_(2,12)_ = 14.280, *p* *=* 0.001; post-hoc, *p* = 0.007 for stressed vs. nonstressed, Fig. 1c). The mean mPFC volume in the stressed + running rats was 32.70 ± 3.22 mm^3^, which was significantly larger than that in stressed rats (post-hoc, *p* < 0.001 for stressed + running vs. stressed, Fig. 1c).

### Running exercise increased the expression of MBP in the mPFC of CUS rats

**Fig. 3:**
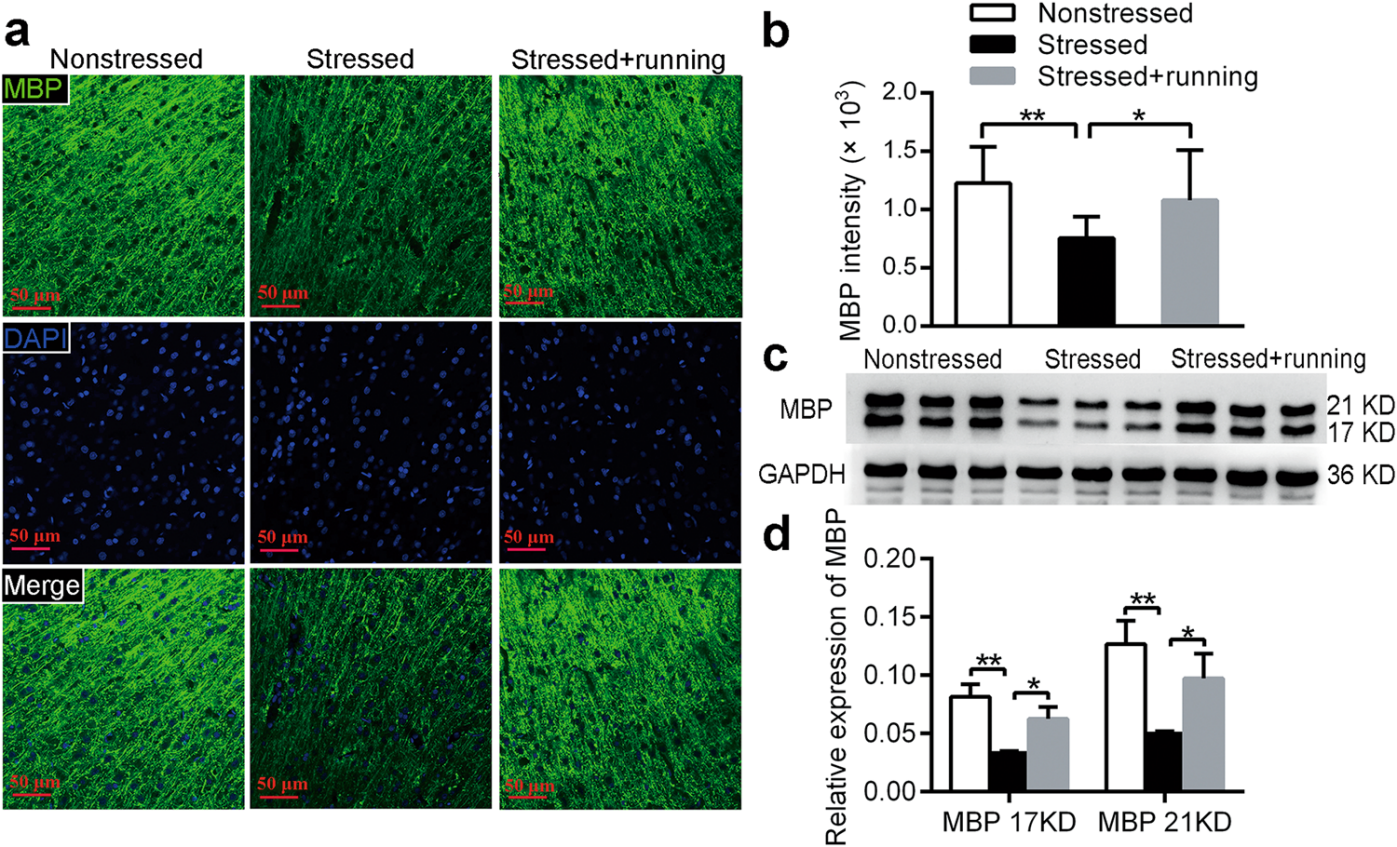
The effects of running exercise on the expression of MBP in the mPFC of CUS rats. **a** Immunofluorescent staining with anti-MBP antibody in the mPFC of the nonstressed group, stressed group and stressed + running group. MBP: green, DAPI: blue. Scale bar = 50 μm. **b** Quantification of MBP intensity in the mPFC of nonstressed rats, stressed rats and stressed + running rats (mean ± SD, *n* = 5 per group). **c** The protein expression of MBP in the mPFC from each group of rats was detected using western blot. **d** Semiquantitative analyses of the protein level of MBP in the mPFC from each group (mean ± SD, *n* = 8 per group). *p < 0.05, **p < 0.01. mPFC medial prefrontal cortex.

To assess the effect of running exercise on mature oligodendrocytes and the myelin sheath, immunofluorescence and western blotting were used to detect the expression of MBP in the mPFC in each group of rats. In the present study, the MBP intensity in the stressed group was significantly decreased compared to that in the nonstressed group (one-way ANOVA *F*_(2,12)_ = 14.238, *p* *<* 0.001; post-hoc, *p* *<* 0.001 for stressed vs. nonstressed Fig. 3a, b), and the MBP intensity in the stressed + running group was significantly higher than that in the stressed group (post-hoc, *p* = 0.001 for stressed vs. nonstressed, Fig. 3a, b). Meanwhile, the protein levels of MBP in the mPFC were also decreased in the stressed group compared to the nonstressed group (17 KD: one-way ANOVA *F*_(2,21)_ = 24.25, *p* *=* 0.001; post-hoc, *p* < 0.001; 21 KD: one-way ANOVA *F*_(2,21)_ = 15.676, *p* *=* 0.004; post-hoc, *p* = 0.001 for stressed vs. nonstressed, Fig. 3c, d), and the protein levels of MBP in the stressed + running group were significantly higher than those in the stressed group (17 KD: post-hoc, *p* = 0.006; 21 KD: post-hoc, *p* = 0.014 for stressed + running vs. stressed, Fig. 3c, d). These results indicated that there was a significant decrease in myelination in the mPFC after CUS and that running exercise improved myelination in the mPFC of the CUS rats.

### Running exercise increased oligodendrocyte differentiation in the mPFC of CUS rats

The enhanced myelination in the mPFC of stressed rats by running exercise could be due to an increased proliferation or differentiation of OPCs or a combination of the two. To verify this hypothesis, we quantified cells that were immunoreactive for NG2, a marker for OPCs, those that were immunoreactive for CNPase, a marker for mature oligodendrocyte, possibly also immature oligodendrocytes and those that were immunoreactive for Olig2, an oligodendrocyte lineage marker^43^ in the mPFC.

**Fig. 4:**
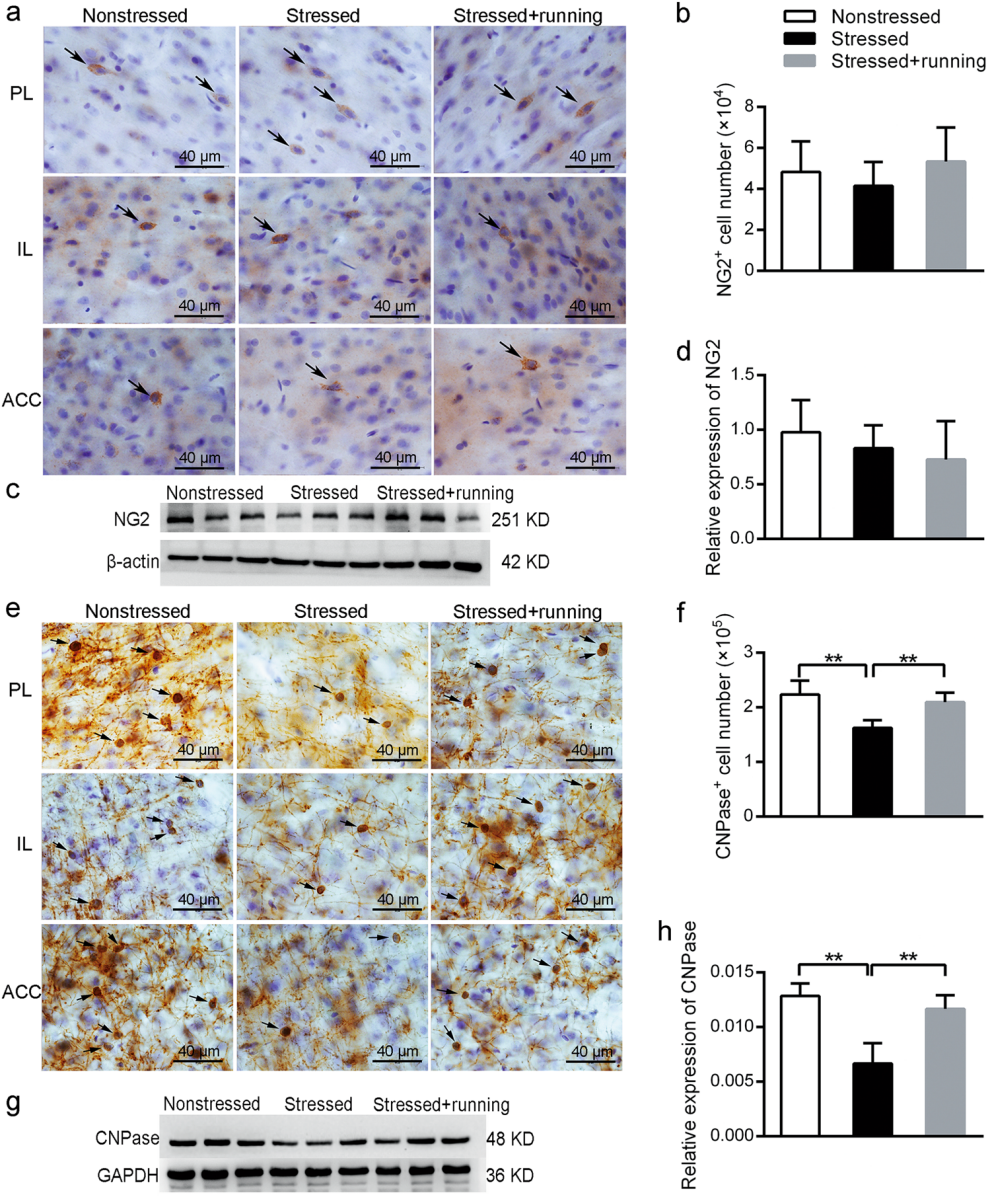
The effects of running exercise on the total NG2^+^ cell numbers and the total CNPase^+^ cell number in the mPFC of CUS rats. **a** Representative pictures of immunohistochemical staining with anti-NG2 antibody in each subregion of the mPFC of the nonstressed rats, stressed rats and stressed + running rats. Scale bar = 40 μm. **b** Total NG2^+^ cell number in the mPFC in the nonstressed group, stressed group and stressed + running group (mean ± SD, *n* = 5 per group). **c** The protein expression of NG2 in the mPFC of each group rats was detected using western blot. **d** Semiquantitative analyses of the protein level of NG2 in the mPFC from each group (mean ± SD, *n* = 8 per group). **e** Representative pictures of immunohistochemical staining with anti-CNPase antibody in each subregion of the mPFC from the nonstressed rats, stressed rats and stressed + running rats. Scale bar = 40 μm. **f** Total CNPase^+^ cell number of mPFC in the nonstressed rats, stressed rats and stressed + running rats (mean ± SD, *n* = 5 per group). **g** The protein expression of CNPase in the mPFC of each group of rats was detected using western blot. **h** Semiquantitative analyses of the protein level of CNPase in the mPFC from each group (mean ± SD, *n* = 8 per group). **p < 0.01. PL prelimbic, IL infralimbic, ACC anterior cingulate cortices.

Representative pictures of immunohistochemical staining with anti-NG2 antibody are shown in Fig. 4a. Using a stereological method combined with immunohistochemistry, the mean total number of NG2^+^ cells in the mPFC were 4.83 (±1.49) × 10^4^ in the nonstressed rats, 4.15 (±1.17) × 10^4^ in the stressed rats and 5.34 (±1.66) × 10^4^ in the stressed + running rats, with CE values of 5.7%, 6.7% and 5.5%, respectively. There were no significant differences in the total number of NG2^+^ OPC cells in the mPFC among the three groups (one-way ANOVA *F*_(2,12)_ = 0.844, *p* *=* 0.454, Fig. 4b). Additionally, via western blot analysis, the protein levels of NG2 in the mPFC were detected in each of the three groups and there were no significant differences among the three groups (one-way ANOVA *F*_(2, 21)_ = 0.561, *p* *=* 0.598, Fig. 4c, d).

Representative pictures of immunohistochemical staining with anti-CNPase antibody are shown in Fig. 4e. Using a stereological method combined with immunohistochemistry, the mean total number of CNPase^+^ cells in the mPFC was 2.23 (±0.25) × 10^5^ in the nonstressed rats, 1.62 (±0.14) × 10^5^ in the stressed rats and 2.09 (±0.17) × 10^5^ in the stressed + running rats, with CE values of 4.3%, 4.8% and 4.3%, respectively. These results represented a significant decrease of 27.35% in CNPase^+^ cells in the rat mPFC induced by CUS (one-way ANOVA *F*_(2,12)_ = 13.293, *p* *=* 0.001; post-hoc, *p* < 0.001 for stressed vs. nonstressed, Fig. 4f). However, running exercise significantly reversed the CNPase^+^ oligodendrocyte loss induced by CUS (post-hoc, *p* = 0.003 for stressed + running vs. stressed, Fig. 4f). In addition, the protein levels of CNPase in the mPFC were detected in each of the three groups using western blot analysis. The protein levels of CNPase in the mPFC was also significantly decreased in the stressed group, and the running exercise significantly reversed this decrease (one-way ANOVA *F*_(2,21)_ = 15.419, *p* *=* 0.004; post-hoc, *p* = 0.002 for stressed vs. nonstressed, *p* = 0.006 for stressed + running vs. stressed, Fig. 4g, h).

**Fig. 5:**
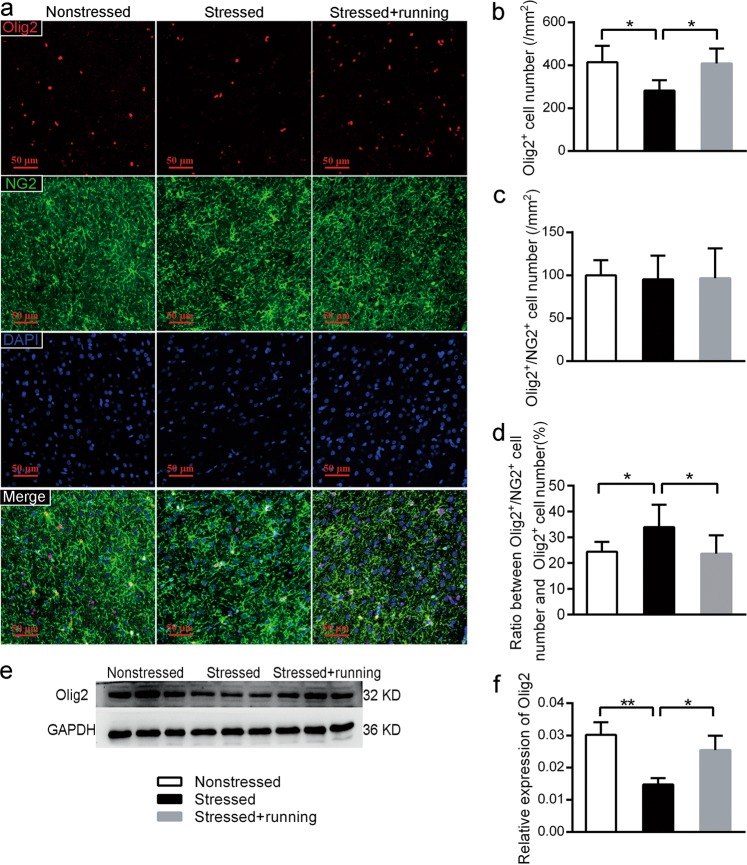
The effects of running exercise on the oligodendrocyte differentiation in the mPFC of CUS rats. **a** Representative pictures of immunofluorescence staining with anti-Olig2 antibody and anti-NG2 antibody in the mPFC from the nonstressed group, stressed group and stressed + running group. Scale bar = 50 μm. **b** Quantification of Olig2^+^ cell number in the mPFC from the three groups of rats after immunofluorescence staining (mean ± SD, *n* = 5 per group). **c** Quantification of Olig2^+^/NG2^+^ cell number in the mPFC from the three groups of rats after immunofluorescence staining (mean ± SD, *n* = 5 per group). **d** The percentage of Olig2^+^/NG2^+^ cells of the Olig2^+^ cells in the mPFC from three groups of rats after immunofluorescence staining (mean ± SD, *n* = 5 per group). **e** The protein expression of Olig2 in the mPFC from each group of rats was detected using western blot. **f** Semiquantitative analyses of the protein level of Olig2 in the mPFC from each group (mean ± SD, *n* = 8 per group). *p < 0.05, **p < 0.01. mPFC medial prefrontal cortex.

Representative pictures of immunofluorescence staining with anti-Olig2 antibody and anti-NG2 antibody are shown in Fig. 5a. After quantifying the Olig2^+^ cells and Olig2^+^/NG2^+^ cells in the mPFC with immunofluorescence, we found that there was a significant decrease in the density of the Olig2^+^ cells (one-way ANOVA *F*_(2,12)_ = 6.532, *p* *=* 0.012; post-hoc, *p* = 0.008 for stressed vs. nonstressed, Fig. 5b) but a significant increase in the ratio between Olig2^+^/NG2^+^ cell number and Olig2^+^ cell number (one-way ANOVA *F*_(2,12)_ = 4.812, *p* *=* 0.029; post-hoc, *p* = 0.024 for stressed vs. nonstressed, Fig. 5d) in the mPFC of the stressed rats compared to the nonstressed rats. However, the stressed + running rats showed a significantly higher density of Olig2^+^ cells (post-hoc, *p* = 0.01 for stressed + running vs. stressed) and a lower ratio between Olig2^+^/NG2^+^ cell number and Olig2^+^ cell number (post-hoc, *p* = 0.07 for stressed + running vs. stressed) in the mPFC than the stressed rats (Fig. 5b, d). Moreover, the protein levels of Olig2 in the mPFC were also decreased in the stressed rats, and the running exercise reversed this decrease (one-way ANOVA *F*_(2,21)_ = 14.506, *p* *=* 0.005; post-hoc, *p* = 0.002 for stressed vs. nonstressed, *p* = 0.011 for stressed + running vs. stressed, Fig. 5e, f). There were no significant differences in the density of the Olig2^+^/NG2^+^ cells in the mPFC among the three groups (one-way ANOVA *F*_(2,12)_ = 0.025, *p* *=* 0.976, Fig. 5c). These results implied that there was a higher percentage of OPCs that did not differentiate in the mPFC in the stressed rats, while the running exercise could promote oligodendrocyte differentiation of CUS rats.

## Discussion

The mPFC has been deemed to be one of most vulnerable areas in MDD^2,3^. Numerous studies have either directly or indirectly shown a decrease in the mPFC volume in both MDD patients and animal models useful for the study of depression^5,6,44^. However, all the data from the previous clinical studies were indirect, and the studies have lacked accurate measurements. In a rodent study, using stereological methods, Noorafshan *et al*. found that the mPFC volume was significantly decreased in the rats exposed to chronic variable stress and antidepressants could restore the stress-induced reduction in the mPFC volume^44^. However, Noorafshan *et al*. did not provide behavioral evidence to validate that the rats in their study presented stress-induced depression-like behaviors. In the present study, rats were exposed to CUS and the SPT was used to evaluate anhedonia, the core symptom of depression. Then, we used the stereological method to estimate the mPFC volume and found that CUS significantly induced mPFC volume loss. Therefore, our results provided accurate quantitative evidence for mPFC volume loss in depression.

Both clinical studies and animal studies have demonstrated that running exercise can have preventive or treatment effects on MDD^23–27,36,45^. Some clinical studies also have reported the beneficial effects of physical exercise on the PFC volume in normal individuals or schizophrenia patients^28–31^. However, there remains a lack of evidence regarding the effects of physical exercise on the PFC volume in depression. In the present study, we found that 6 weeks of running exercise significantly reversed the anhedonia and volume loss in the mPFC of CUS rats. It has been reported that currently remitted participants had a larger PFC than currently depressed participants^6^. Yucel *et al*. also found that an increase in subgenual PFC volume was associated with long-term antidepressant therapy^9^. Thus, the volume of the mPFC is closely related to depression and might be an indicator of the illness burden, medication status and treatment response. Therefore, our present results suggested that running exercise had positive effects on the mPFC volume in the context of depression, which provided new structural evidence for the antidepressant effects of running exercise.

Impaired myelination in the PFC has been reported in MDD patients and depression-like animal models^18,46,47^. In the present study, decreased intensity of MBP in the immunofluorescence results and lower protein expression of MBP in the mPFC of stressed rats also implied a myelination impairment in the mPFC induced by CUS. What causes the impairment in myelination in the mPFC induced by CUS? Mature oligodendrocytes are myelin-forming cells in the central nervous system. Many investigations have suggested that oligodendrocytes may be particularly susceptible to stress-related and corticosterone-induced toxicity^21,48,49^. Additionally, a low density of oligodendrocytes and altered expression of oligodendrocyte-specific gene transcripts in postmortem human subjects and animal models points toward decreased oligodendrocytes in the PFC of depression^21,46,50^. However, using stereological methods and immunohistochemistry, we found that there was no significant difference in the number of NG2^+^ OPCs in the mPFC between nonstressed rats and stressed rats. Our results were different from the results of Birey *et al*.^51^. Birey *et al*. reported that NG2^+^ OPC density in the PFC was reduced in subjects with MDD and in susceptible mice after exposure to chronic social defeat stress^51^. We thought the difference in the quantitative methods might explain the difference. In the study of Birey *et al*., they used two-dimensional quantitative method to obtain the NG2^+^ OPC density in the PFC, and their density result could not directly reflect the total number of the NG2^+^ OPCs in the PFC. In our study, we used three-dimensional stereological methods to quantify the total number of NG2^+^ OPCs in the mPFC, and our results were unbiased and accurate. Oligodendrocyte differentiation is a prerequisite for myelin biogenesis^13^. In the following study, we found that CUS induced significantly decrease in the total number of CNPase^+^ mature and immature oligodendrocytes in the mPFC. In addition, using immunofluorescence, we found that CUS might also have caused an impairment in oligodendrocyte differentiation in the rat mPFC because there was a significant decrease in the density of the Olig2^+^ cells but a significant increase in the ratio between Olig2^+^/NG2^+^ cell number and Olig2^+^ cell number in the mPFC in the stressed group when compared with the nonstressed group. Therefore, we speculated that reducing the OPC differentiation in the mPFC might be one of the main reasons that the CUS impairs myelination, which was similar to the previous study^52^.

Previous studies have indicated that promoting adult myelination is a potential strategy for reversing depressive-like behavior^22^. Meanwhile, interventions such as electroconvulsive therapy alleviated depressive-like behaviors and enhanced proliferation and survival of OPCs in the rat PFC and amygdala^53,54^. In the present study, 6 weeks of running exercise significantly increased the number of CNPase^+^ oligodendrocytes and the density of Olig2^+^ oligodendrocytes, reduced the ratio between Olig2^+^/NG2^+^ oligodendrocytes and Olig2^+^ oligodendrocytes in the mPFC of the CUS rats, and increased the protein expression of MBP, CNPase and Olig2. These results indicated that 6 weeks of running exercise enhanced the differentiation and probably proliferation of oligodendrocytes and promoted myelination in the mPFC in CUS rats. We speculated that the beneficial effects of exercise on oligodendrocytes might be an important cellular basis for running exercise as an intervention to treat depression. Interestingly, chronic treatment with the antidepressant fluoxetine showed no changes in oligodendrocyte proliferation in the prelimbic cortex or in oligodendrocyte-related gene expression in the cingulate cortex and amygdala of nonstressed rats or mice^55,56^. Our previous studies also found that fluoxetine did not ameliorate the CUS-induced myelin impairment in the white matter of rats^57^. These results suggested that oligodendrocyte changes may not be involved in the mechanism of particular pharmacological antidepressant treatments. Therefore, we propose that running exercise might be a good option that combines with pharmacological antidepressant treatment to reinforce the antidepression effects.

Although many studies have affirmed the positive effects of physical exercise on oligodendrocytes in many brain regions both in normal animals and animal models of disease^32^, there is no unified view of whether exercise could promote either OPC proliferation or oligodendrocyte differentiation, or a combination of both. In the present study, 6 weeks of running exercise showed positive effects on oligodendrocyte differentiation, myelination and probably proliferation in the mPFC of CUS-induced depression-model rats. These results in the present study were similar to some previous studies to some extent. For instance, training on a running wheel could stimulate both OPC proliferation and oligodendrocyte differentiation in the corpus callosum of normal mice^58^. Similarly, voluntary running triggered OPC proliferation, oligodendrocyte differentiation and myelination in the cerebellum to prolong the lifespan of ataxic cerebellum mice^59^. However, Mandyam *et al*. suggested that 4 weeks of voluntary running just enhanced mPFC gliogenesis in normal animals, and the OPCs were the most affected cell type^60^. In addition, Simon *et al*. found that a two-week period of free access to running wheels suppressed the proliferation but promoted the differentiation of oligodendrocytes in the motor and somatosensory cortices of normal mice^33^. One possible reason for the different effects of running exercise on the oligodendrocytes might be that the brain regions and animal models studied in these studies were different. It has been widely accepted that in healthy bodies, physical skills can result in increases in myelination in a number of associative white matter regions^61,62^, with similar findings demonstrated in rodents^32^. Therefore, we hypothesize that in healthy bodies, running exercise could mainly promote OPC to exit the cell cycle, i.e., promote the differentiation of OPC and induce the myelination in the areas directly associated with running exercise, such as motor and somatosensory cortices, which was proven by Simon *et al*.^33^. In the areas indirectly associated with running exercise, such as the mPFC, running exercise could mainly promote OPC proliferation to increase the reserved oligodendrocytes. However, in some diseases with demyelination or impaired myelination, physical exercise could mainly trigger differentiation and myelination in the affected brain regions, therefore resulting in treatment-related effects, as in many previous studies^36,59,63,64^ and the present study. Another possible reason for the different effects of running exercise on oligodendrocytes might be the different forms of running exercise. It has been demonstrated that forced and voluntary exercise differentially affected brain and that forced exercise induced more BrdU^+^ cells in the dentate gyrus than voluntary exercise^65^. Hayes *et al*. found that forced, but not voluntary, exercise effectively induced neuroprotection in stroke-model rats^66^. As forced and voluntary exercise are inherently different, the two forms of exercise are likely producing differential effects on brain, whereas the studies in the normal animal models mentioned above all provided voluntary exercise. In the present study, we are the first to study the effects of forced exercise on oligodendrocytes in the mPFC of CUS rats. In the future, whether forced and voluntary exercise have different effects on oligodendrocytes in the brain with or without disorders should be studied.

Depression is characterized by its high incidence and serious harm^1^, but the effectiveness of the current antidepressant methods is very limited^67^. Physical activity has been consistently reported as reducing the risk of depression and having antidepressant effects and its antidepressant mechanisms are still being extensively discussed^68^. If the key target molecules or structures of physical activity antidepressant could be found, it might provide a new idea for clinical treatment of depression or enhancing the effectiveness of current antidepressants. Myelin sheath change in the mPFC has been recognized as one of the pathological changes in depression^18,46,47^. In our present study, we provide further evidence for the pathological changes in the oligodendrocytes and myelin sheaths of the mPFC in depression. More importantly, we found that running exercise could reverse depressive-like behaviors and volume loss in the mPFC and promote oligodendrocyte differentiation and myelination of the mPFC of the CUS-induced rat model of depression. Our current findings might provide structural bases for the exercise-induced treatment of depression and provide structural basis for developing new treatments for depression.

## Supplementary information

Supplementary Materials and Methods

Fig S1

Table S1
