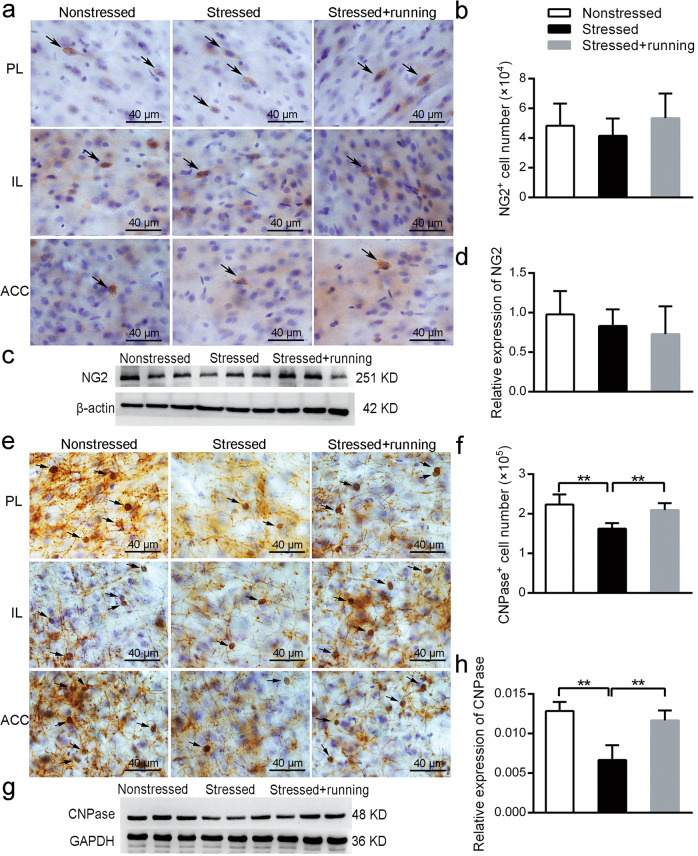# Correction to: Running exercise protects oligodendrocytes in the medial prefrontal cortex in chronic unpredictable stress rat model

**DOI:** 10.1038/s41398-023-02465-8

**Published:** 2023-05-09

**Authors:** Yanmin Luo, Qian Xiao, Jin Wang, Lin Jiang, Menglan Hu, Yanhong Jiang, Jing Tang, Xin Liang, Yingqiang Qi, Xiaoyun Dou, Yi Zhang, Chunxia Huang, Linmu Chen, Yong Tang

**Affiliations:** 1 Laboratory of Stem Cells and Tissue Engineering, Chongqing Medical University, Chongqing, 400016 People’s Republic of China; 2 Department of Physiology, Chongqing Medical University, Chongqing, 400016 People’s Republic of China; 3 Department of Radioactive Medicine, Chongqing Medical University, Chongqing, 400016 People’s Republic of China; 4 Department of Histology and Embryology, Chongqing Medical University, Chongqing, 400016 People’s Republic of China; 5 Institute of Life Science, Chongqing Medical University, Chongqing, 400016 People’s Republic of China

Correction to: *Translational Psychiatry* 10.1038/s41398-019-0662-8 published online 28 November 2019

The original version of this article contained an error. In the original file of Fig. 4g, the loading controls were accidentally mirror-image changed. The loading controls of Fig. 4g should be the same as the loading controls of Fig. 3c, because in the western blotting experiment, the two proteins came from the same piece of separation glue and shared the same loading controls. The authors apologize for the error. The original article has been corrected.