# Supplementary material for: Running exercise protects oligodendrocytes in the medial prefrontal cortex in chronic unpredictable stress rat model
Source: Transl Psychiatry. 2019 Nov 28;9:322. doi: 10.1038/s41398-019-0662-8 (PMC6882819; doi:10.1038/s41398-019-0662-8)
Supplement: Supplementary file 1 — Supplementary Materials and Methods [file 41398_2019_662_MOESM1_ESM.docx]

**Materials and methods**

**Animals**

Sixty male Sprague-Dawley rats (Chongqing Medical University, Chongqing, China) were housed under a 12-h light/12-h dark cycle at a constant temperature (22 °C) with free access to food and water except when animals were subjected to stressors during the CUS procedure. Rats were allowed 2 weeks to acclimate before use in the experiments. Then, all rats were randomly divided into a nonstressed group (n = 23), stressed group (n = 17) and stressed + running group (n = 20). Following 5 weeks of CUS and 6 weeks of running exercise, the behavioral experiments and tissue processing were performed with 19- to 20-week-old rats. During the animal experiment, the animals in each group were treated by the investigators without blinding. All experiments were performed in accordance with the guidelines established by the National Institutes of Health Guide for the Care and Use of Laboratory Animals (NIH publication no. 85-23) and were approved by the Animal Care Committee of the Chongqing Medical University.

**Chronic unpredictable stress (CUS) paradigm**

For CUS, the rats in the stressed group and stressed + running group were housed with one rat per cage and exposed to two to three stressors per day for 5 weeks. The stressors included an electric shock foot (current intensity 1.0-1.5 mA, 2 min), tail clamp (1 min), cold water bath (4°C for 5 min), hot water bath (45 °C for 5 min), cage rotation (20 min), restraint (2 h), noise (85 decibel for 4 h), food or water deprivation overnight, empty bottles (2 h after water deprivation), light on overnight, light off during day (3 h), odor overnight, damp bedding (12 h), stroboscope overnight or tilted cage overnight (45°)^1, 2^. All stressors were randomly interspersed throughout the stress period. The rats in the nonstressed group were not exposed to CUS stimuli and were housed in normal conditions with 5 rats per cage.

**Treadmill running exercise**

After the CUS intervention, rats in the stressed + running group were scheduled for treadmill running exercise using a horizontal motorized treadmill for 6 weeks, 5 days per week, 20 min per day. During the first week, the rats ran at a speed of 10 m/min on the first day, followed by an increase of 2 m/min per day. For the remaining 5 weeks, the running speed was maintained at 20 m/min. This treadmill running pattern has been used successfully in our previous studies ^1, 2^.

During the days of running exercise, the rats in the stressed group and stressed + running group remained housed with one rat per cage but without exposure to the CUS stimuli, and rats in the nonstressed group were still housed in normal conditions with 5 rats per cage.

**Body weight**

The body mass of the rats in each group was recorded during the same time frame each week.

**Sucrose preference test (SPT)**

The animals were first trained to consume a 1% sucrose solution during a period of acclimation. During the training, the rats were housed with one rat per cage and habituated for 48 h to two bottles in their home cages; two bottles of 1% sucrose solution during the first 24 h, and a bottle of 1% sucrose solution and a bottle of water during the next 24 h. The positions of bottles were counterbalanced across the left or right side of the testing cages. Following training, the sucrose preference of each group of rats was tested. The intake of water and a 1% sucrose solution, assessed by weight, were measured over 24 h. Sucrose preference was calculated as the ratio of sucrose solution consumption to total water consumption. SPT was assessed during the same time frame each week.

**Elevated plus maze test**

The elevated plus maze test was performed in the last week of running exercise. The animal was placed at the intersection of the four arms (i.e., the center area), and behavior was recorded for 5 min using a camera. The number of open and closed arm entries along with the time spent in each arm were recorded. An arm entry was defined as 50% of the body being positioned within the arm. The apparatus was cleaned with 70% ethyl alcohol after each trial to remove permeated odors from previous animals.

**Perfusion and tissue processing**

During the following processes, all the experiments and data analysis were performed blind to treatment conditions. After the behavioral testing, under deep anesthesia with an i.p. injection of 1% (4 mL/kg body weight) sodium pentobarbital, 5 rats from each experimental group were randomly selected and perfused transcardially with fixative (4% paraformaldehyde in 0.1 M phosphate-buffered salin (PBS), pH 7.4). Brains were removed and placed in fixative. The brains were split into two hemispheres by a midsagittal section and processed for stereology. The right or left hemisphere from each rat was sampled at random and dehydrated in sucrose dilutions of 10% (24 h), 20% (24 h), 30% (24 h) and 30% (24 h). Then, the hemispheres were coronally sectioned into 60 μm sections on a cryostat microtome (CM1860, Leica). The sections were kept in anatomical series. From the sections containing the mPFC, every 6th section was sampled in a systematic-random manner with 15 sections per series on average. In the end, 6 sets of sampled sections were acquired.

**Cresyl violet staining, region delineation and volume estimation**

One set of sampled sections was randomly chosen. The sampled sections were stained with cresyl violet to help delineate the boundary of the mPFC. Briefly, the sections were placed onto the slides and dried in air for at least for 2 h. Then, the slides were washed with double-distilled water for 2 min and stained with preheated cresyl violet solution (C0117, Beyotime Biotechnology, China) for 40 min at 45 °C. The slides were washed in double-distilled water 3 times. Then, the slides were sequentially immersed in 95% ethanol 4 times (1 min per time), 100% ethanol 3 times (30 s per time) and xylene 3 times (5 min per time). Finally, the sections were mounted with neutral gum.

The mPFC of rats is composed of prelimbic (PL), infralimbic (IL) and anterior cingulate (ACC) cortices ^3^. Under a low-magnification objective lens (4×), the boundary of the mPFC was delineated according to the description in Cerqueira et al. ^3^ and the atlas of Paxinos and Watson ^4^ (Fig. 1a). The total volume of the mPFC was measured using Cavalieri’s principle ^1^. Briefly, a transparent counting grid with an area of 0.205 mm^2^ per grid point was placed at random on each picture, and the points located in the mPFC were counted (Fig. 1b). Then, the mPFC volume, *V_mPFC_*, was calculated according to Cavalieri’s principle ^1^.

| *V_mPFC_* = *t × a(p) × ∑P*， (1) |
| --- |

where t is the distance between two adjacent tissue sections (360 μm), a(p) is the area associated with each grid point (0.205 mm^2^), and ∑P is the total number of grid points located in the mPFC per rat.

**Immunohistochemistry and stereological cell counting**

Two separate sets of serial sections containing the mPFC from each group of rats were chosen and immunoreacted with anti-NG2 antibody and anti-CNPase antibody for the stereologic analyses of the total numbers of OPCs and mature oligodendrocytes in the mPFC. Free-floating sections were treated first with 3% hydrogen peroxide for 25 min to block endogenous peroxidase activity and with saline sodium citrate in a boiling water bath for 30 min for antigen retrieval. Then, the sections were blocked with 10% goat serum and 1% fetal bovine serum at 37 °C for 2 h and incubated with the primary antibodies at 4 °C for 60 h. Primary antibodies included mouse anti-NG2 antibody (1:1000; ab50009, Abcam, USA) and mouse anti-CNPase antibody (1:1000; ab6319, Abcam, USA). Sections were then incubated in the secondary antibody solution (biotinylated anti-mouse IgG; SP9002, Beyotime Biotechnology, China) for 3 h at 37 °C and incubated with avidin-biotin peroxidase complex for 2 h at 37 °C. In the procedures above, all the wash buffers and diluents were 0.3% Triton X-100 and 0.1 % Tween diluted in 0.01 M PBS. Diaminobenzidine (DAB; ZLI-9032, ZSGB-BIO, China) was used as a chromogen. The floating sections were placed onto the slides and stained with Mayer’s hematoxylin to label nuclei. After being washed in double-distilled water 3 times, the sections were dehydrated by sequential immersion in gradient ethanol, transparented in xylene and mounted with neutral gum.

The optical fractionator ^5^ was used to estimate the total numbers of NG2^+^ and CNPase^+^ cells in the mPFC. A contour was traced at 4× magnification around the mPFC according to the adjacent sections with cresyl violet staining. The counting fields were sampled from the delineated region of the sections with an area sampling fraction (asf) of 15% for counting NG2^+^ cells and 8% for counting CNPase^+^ cells. A 100× oil immersion lens with a numerical aperture of 1.4 was used to count the cells in each of the sampled counting fields. A 3-µm ‘guard zone’ depth was set at the top surface of the sections. Counting was performed with the optical disector through a depth of 15 µm. In the present study, the optical disector height divided by the overall average section thickness yields the section thickness sampling fraction, 15/34.2 on average in counting NG2^+^ and 15/32.3 on average in counting CNPase^+^ cells.

Based on the above parameters set in the stereology analysis system, a three-dimensional probe (optical disector) was used to directly count the number of positive cells in a known fraction of the volume of the mPFC. When the microscopic field of vision was clearly focused for the first time, its location on the Z axis was set to 0. Then, as the microscope was adjusted, the positive cells within the guard zones were not counted. Thereafter, as the Z axis moved below the guard zones, if the nuclei of positive cells came into focus and did not touch the forbidden lines of the unbiased counting frame, those cells were counted (Fig. S1a, b). On average, 269 NG2^+^ cells and 647 CNPase^+^ cells were sampled per animal. Once the analysis was completed, the total numbers of NG2^+^ cells and CNPase^+^ cells in the mPFC were calculated by multiplying the total numbers of positive cells counted in the sampled volume of tissue by the reciprocal of the sampling fractions ^5^.

**Immunofluorescence, image acquisition and analysis**

From one set of sampled sections, every second section was sampled again in a systematic-random fashion resulting in two series of sections with 8 sections per series on average. The two series of sections were labeled with Olig2/NG2 antibodies against the OPCs and MBP antibodies against the myelin sheaths. The floating sections were blocked with 10% goat serum, 1% fetal bovine serum, and 2.5% donkey serum for 2 h and incubated with the primary antibodies at 4 °C for 60 h. Sections were incubated with secondary antibodies for 2 h at 37 °C. Primary antibodies included rabbit anti-Olig2 (1:500; ab109186, Abcam, USA), mouse anti-NG2 (1:500; ab50009, Abcam, USA) and mouse anti-MBP (1:500; ab62631, Abcam, USA). Secondary antibodies included AlexaFluor-488-conjugated secondary antibodies to mouse and AlexaFluor-549-conjugated secondary antibodies to rabbit (1:100; Abbkine, USA). Fluorescent images were collected on a laser confocal scanning microscope (A1R, Nikon, Japan) with the excitation wavelengths appropriate for AlexaFluor-488 (488 nm), AlexaFluor-549 (549 nm) or DAPI (380 nm). Sixteen randomly chosen fields were used for cell counting in each rat. Quantification of MBP immunostaining was performed using NIS-Elements 4.3.

**Western blotting**

Rats (8 per group) were randomly chosen and anesthetized with sodium pentobarbital, and mPFC tissue was rapidly removed and frozen. Frozen samples were homogenized, and proteins were extracted using a RIPA lysis buffer with 1% PMSF solution (Beyotime Biotechnology, China). After protein determination using a BCA kit (Beyotime Biotechnology, China), sodium dodecyl sulfate-polyacrylamide gel electrophoresis and western blotting were carried out. The following antibodies were used at the indicated dilutions: rabbit anti-Olig2 (1:1000; ab109186, Abcam, USA), mouse anti-MBP (1:1000; ab62631, Abcam, USA), mouse anti-CNPase (1:1000; ab6319, Abcam, USA) and mouse anti-NG2 (1:1000; ab50009, Abcam, USA). Mouse anti-β-actin (1:1000; Zoonbio Biotechnology, China) and rabbit anti-GAPDH (1:1000; Boster Biological Technology, China) were used as internal controls for the concentration of protein loaded. Immunoreactive proteins were detected using HRP-conjugated goat anti-mouse (1:2000; Boster Biological Technology, China) or goat anti-rabbit antibody (1:2000; Boster Biological Technology, China) and visualized using an ECL detection kit (Beyotime Biotechnology, China). Quantification of band intensity was analyzed using Image Lab software (version 5.2.1).

**Statistics**

All statistical analyses were performed using SPSS 19.0. The Shapiro-Wilk test was used to evaluate whether the data were normally distributed. The Levene’s test was used to evaluate whether the variances were similar among the groups. Then the data from the body weight were analyzed using repeated measures analysis of variance (AVOVA). For the remaining data, if they were normally distributed and had similar variance among groups, one-way ANOVA followed by LSD post hoc test were used for analysis; otherwise, Kruskal-Wallis test was adopted for analysis. A p value < 0.05 was adopted as the level of significance for all the analyses. The coefficient of error (CE) of the mPFC volume was calculated according to Gundersen et al. ^6^. The CE of the NG2^+^ cells and CNPase^+^ cells in the mPFC were calculated according to the method described by Schmitz and Hof ^7^. Sample size for each experiment was chosen based on previous experience and aimed to detect at least a *p* < 0.05 in the different tests applied. No animals were excluded from the current study.

**References**

1. Xiao Q, *et al*. Exercise protects myelinated fibers of white matter in a rat model of depression. *The Journal of comparative neurology* 2018; **526**(3)**:** 537-549.

2. Tang J, *et al*. The effects of running exercise on oligodendrocytes in the hippocampus of rats with depression induced by chronic unpredictable stress. *Brain research bulletin* 2019; **149:** 1-10.

3. Cerqueira JJ, *et al*. Morphological correlates of corticosteroid-induced changes in prefrontal cortex-dependent behaviors. *The Journal of neuroscience : the official journal of the Society for Neuroscience* 2005; **25**(34)**:** 7792-7800.

4. Paxinos G, Watson C. *The rat brain in stereotaxic coordinates: hard cover edition*. Elsevier, 2006.

5. West MJ, Slomianka L, Gundersen HJ. Unbiased stereological estimation of the total number of neurons in thesubdivisions of the rat hippocampus using the optical fractionator. *The Anatomical record* 1991; **231**(4)**:** 482-497.

6. Gundersen HJ, Jensen EB, Kieu K, Nielsen J. The efficiency of systematic sampling in stereology--reconsidered. *Journal of microscopy* 1999; **193**(Pt 3)**:** 199-211.

7. Schmitz C, Hof PR. Recommendations for straightforward and rigorous methods of counting neurons based on a computer simulation approach. *Journal of chemical neuroanatomy* 2000; **20**(1)**:** 93-114.

**Figure legends:**

**Fig. S1** Illustration of the method used to count the NG2^+^ cells (**a**) and CNPase^+^ cells (**b**) via optical disector. Bar = 10 μm. Briefly, a 100× oil immersion lens with a numerical aperture of 1.4 was used to count the cells in each of the sampled counting fields. A 3-µm ‘guard zone’ depth was set at the top surface of the sections. Counting was performed with the optical disector through a depth of 15 µm. The positive cells with clear nucleoli inside the counting frame or only touching the green lines, but not touching the red lines, are counted, as indicated by arrows.
