# Supplementary figures and images for: Running exercise protects oligodendrocytes in the medial prefrontal cortex in chronic unpredictable stress rat model
Source: Transl Psychiatry. 2019 Nov 28;9:322. doi: 10.1038/s41398-019-0662-8 (PMC6882819; doi:10.1038/s41398-019-0662-8)

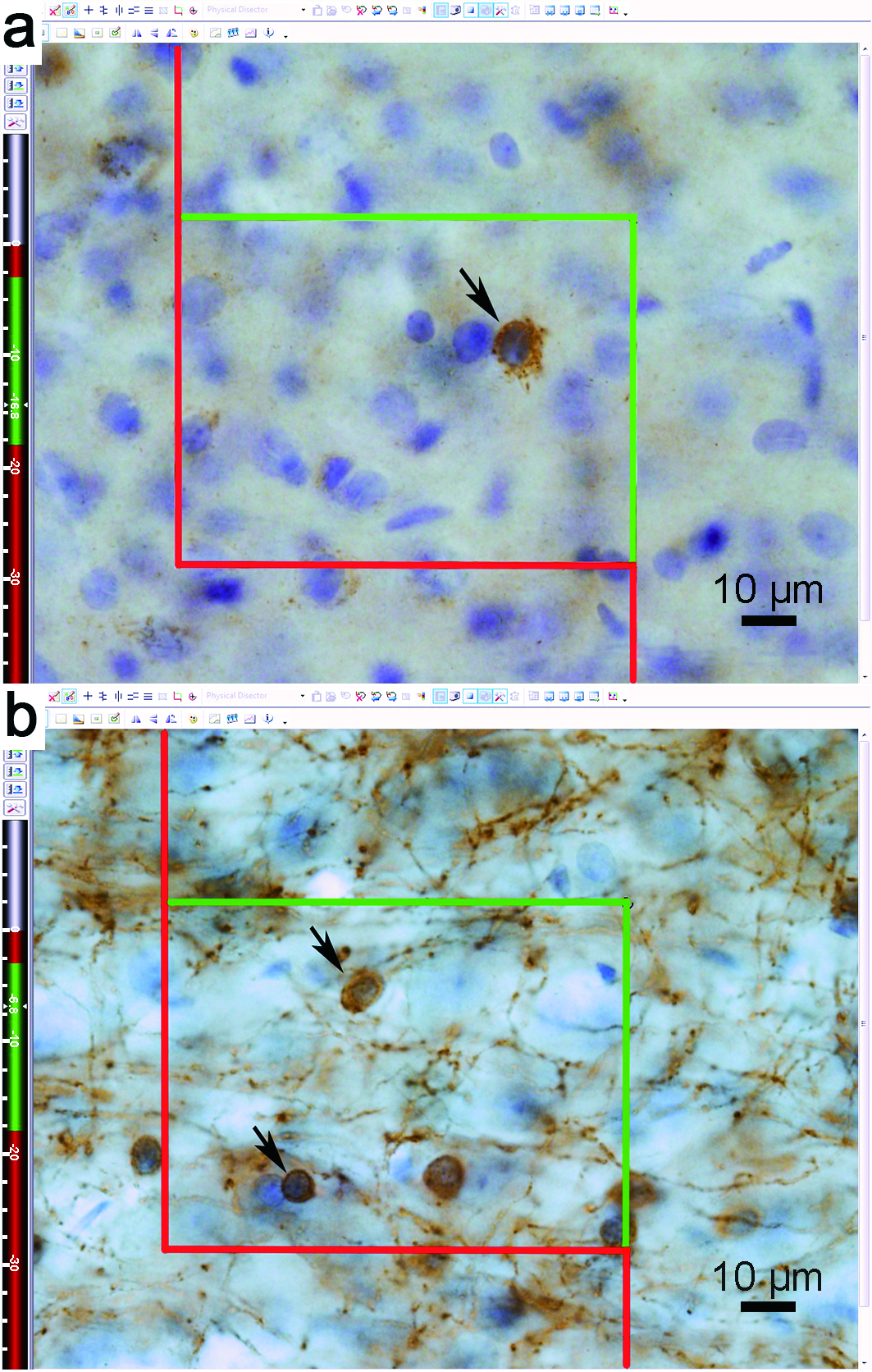

Supplement: Supplementary file 2 — Fig S1 [file 41398_2019_662_MOESM2_ESM.tif]
