# Supplementary material for: Running exercise protects oligodendrocytes in the medial prefrontal cortex in chronic unpredictable stress rat model
Source: Transl Psychiatry. 2019 Nov 28;9:322. doi: 10.1038/s41398-019-0662-8 (PMC6882819; doi:10.1038/s41398-019-0662-8)
Supplement: Supplementary file 3 — Table S1 [file 41398_2019_662_MOESM3_ESM.docx]

Table S1. The results of the elevated plus maze test

|  | Nonstressed  (n = 23) | Stressed  (n =17) | Stressed + running  (n =20) | *p* value |
| --- | --- | --- | --- | --- |
| Number of open arm entries | 1 (1) | 1 (1) | 1.65 ± 1.09 | Kruskal-Wallis,  χ^2^ = 2.764, df = 2, *p* = 0.251 |
| The time in open arms (s) | 12.15 (33.00) | 14.40 (17.60) | 27.13 ± 21.38 | Kruskal-Wallis,  χ^2^ = 2.561, df = 2, *p* = 0.278 |
| Number of close arm entries | 8.86 ± 3.71 | 7.94 ± 3.42 | 8.35 ± 3.73 | one-way ANOVA, F_(2,57)_ = 0.308, *p* = 0.736 |
| The time in close arms (s) | 188.36 ± 51.58 | 204.92 ± 47.29* | 148.30 ± 52.93 | one-way ANOVA, F_(2,57)_ = 6.079, *p* = 0.004 |
| Number of arm entries | 10.41 ± 4.03 | 9.00 ± 3.62 | 10.00 ± 4.03 | one-way ANOVA, F_(2,57)_ = 0.613, *p* = 0.545 |
| The percentage of the number of open arm entries（%） | 10.56 (13.44) | 11.99 ± 8.62 | 17.14 ± 12.05 | Kruskal-Wallis,  χ^2^ = 1.625, df = 2, *p* = 0.444 |
| The percentage of the time in open arms（%） | 4.05 (11.00) | 4.80 (5.85) | 9.04 ± 7.13 | Kruskal-Wallis,  χ^2^ = 2.561, df = 2, *p* = 0.278 |

When the data are normally distributed, the values are presented as mean ± SD; when the data are not normally distributed, the values are presented as median (quartile spacing). * indicates *p* = 0.002, when the time in close arms (s) of stressed + running group is compared with that of the stressed group.
